# The effect of neuroimmune signalling on drug addiction

**DOI:** 10.3389/fphar.2026.1830998

**Published:** 2026-07-06

**Authors:** Qianzhao Chen, Haoran Chi, Shuang Liu, Yue Zhou

**Affiliations:** 1 Department of Pharmacy, Liang jiang Hospital of Chongqing Medical University, Chongqing, China; 2 Department of Pharmacy, Xindu District People’s Hospital of Chengdu, Chengdu, China

**Keywords:** blood–brain barrier, cocaine, drug addiction, neuroimmune signalling, neuroinflammation

## Abstract

The relationship between neuroimmune processes and drug addiction is currently unclear, although long-term drug use affects neuroimmune signalling. Currently, how neuroinflammation and neuroimmune signalling are linked to addiction is unclear. Therefore, the purpose of this review is to elucidate the roles of neuroimmune signalling, its interaction with neuroinflammation resulting from long-term drug use, and the possible influence of this interaction on the use of pharmacotherapeutic approaches for treating drug addiction. In this review, we address the roles of chemokines and cytokines in the rewarding and reinforcing effects of psychostimulants. Additionally, we provide an overview of novel anti-inflammatory therapeutic strategies that differ from conventional strategies by focusing on cytokines and chemokines. Understanding the role that neuroimmune signalling plays in drug addiction may help researchers develop innovative strategies for addressing this serious social problem.

## 1 Introduction

Inflammation involves a complex cascade of processes that cause both tissue damage and repair and is classically classified as acute or chronic. While pathologically distinct, these forms of inflammation share four common cellular and molecular hallmarks: elevated levels of certain cytokines and chemokines, activation of macrophages, recruitment of leukocytes, and local tissue damage (Estes and McAllister, 2014). When an inflammatory process occurs in the central nervous system (CNS), it is referred to as “neuroinflammation”. No formal definition exists for this term, but it was historically associated with immune-driven pathology and disruption of the blood‒brain barrier (BBB) in conditions such as stroke, trauma, or infection. The concept was subsequently expanded to include autoimmunity (Kölliker-Frers et al., 2021). Proinflammatory cytokine production, glial activation, and secondary cell death, along with immune cell infiltration, recapitulate the four classical hallmarks of peripheral inflammation (Estes and McAllister, 2014). However, recent studies have broadened the definition of neuroinflammation, classifying processes with single or multiple soft hallmarks of inflammation as inflammatory and considering the existence of different degrees of neuroinflammation. This change has led to the term “neuroinflammation” lacking a universally accepted meaning, and multiple scenarios that are different from the classic concept are now increasingly encompassed under the same umbrella term (Aguzzi et al., 2013; Galea and Graeber, 2023). This broadening of the neuroinflammation concept has direct implications for the drug addiction field. Many of the cellular and molecular mediators involved in neuroinflammation, such as cytokine, glia, and immune signalling pathways, also participate in the pathological processes underlying drug addiction.

Some people with drug addiction, a chronic and complicated disease, develop problematic drug use and use-related disorders after periods of episodic drug use. These disorders are likely to involve periods of drug use followed by abstinence and eventual relapse (Koob and Volkow, 2010; Nestler and Lüscher, 2019). Psychoactive substances are traditionally recognized based on their neurotoxic effects on glutamatergic and monoaminergic systems (Nestler and Lüscher, 2019). These substances also promote neuroinflammation, which appears to contribute to the development of problematic drug use and addiction (Kohno et al., 2019; Miguel-Hidalgo, 2009).

## 2 Inflammation and drug addiction

Classically activated immune cells, including macrophages, lymphocytes, T cells, and microglia, are primarily responsible for the production of proinflammatory cytokines in the CNS and peripheral nervous system (He et al., 2021; Zhao et al., 2017). According to recent research, substances of abuse exert widespread effects on inflammation. The serum of individuals with psychostimulant addiction contains high levels of proinflammatory cytokines and low levels of anti-inflammatory cytokines (Fox et al., 2012). Similarly, opioids can trigger inflammatory responses in the brain, which can contribute to opioid dependence (Eidson and Murphy, 2019). Additionally, individuals who are addicted to alcohol or nicotine present elevated blood levels of the inflammatory marker C-reactive protein (CRP) (Costello et al., 2013). Among the cannabinoids, low concentrations of delta-9-tetrahydrocannabinol (Δ9-THC) increase the level of the anti-inflammatory cytokine IL-10 in microglia, thereby attenuating the release of interleukin (IL)-1β, IL-6, and IFN-γ (Kozela et al., 2010). Crucially, deactivating immune cells or administering anti-inflammatory medications lowers the rewarding effects of a drug and reduces dependent subjects’ desire to use drugs (Beardsley et al., 2010; Metz et al., 2017). These findings point to the prevalence of proinflammatory reactions associated with various substances of abuse. Furthermore, intricate connections between inflammation in the CNS and peripheral nervous system may contribute to drug addiction. By promoting the production of specific cellular adhesion molecules and increasing BBB permeability, peripheral inflammation can disrupt the BBB (Turkheimer et al., 2021; Vore et al., 2022). This disruption increases the levels of inflammatory signals in the brain by allowing immune cells and cytokines to enter the CNS. Furthermore, some addictive medications might exacerbate this behaviour by directly damaging the BBB (Gonçalves et al., 2017). In conclusion, the relationship between inflammation in the CNS and peripheral nervous system is bidirectional. Neuroinflammation in the CNS can be influenced by peripheral immune cells and cytokines, while peripheral immune cell activity can also be affected by changes in the immunological status of the brain. The development of successful treatments that address the dual effects of addictive substances on the immune system and mental health requires an understanding of these intricate interactions.

**FIGURE 1:**
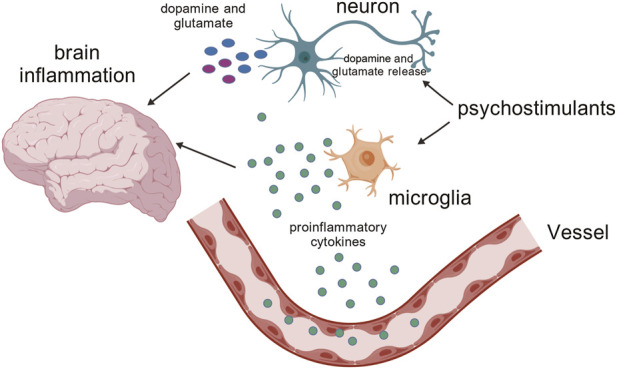
Psychostimulant-induced neuroinflammation. In the brain, psychostimulants can activate microglia and subsequently promote proinflammatory cytokine release. Moreover, the excess dopamine and glutamate released by neurons can promote the expression of inflammatory cytokines to induce neuroinflammation.

Numerous studies have shown that psychostimulants affect cytokine synthesis and release in the CNS (Canedo et al., 2021; Coelho-Santos et al., 2015; Mata et al., 2015; Pianca et al., 2017) (Figure 1). However, the interaction between the central and peripheral immune systems in individuals exposed to psychostimulants is still poorly understood. Notably, BBB disruption is among the defining characteristics of the neurotoxicity of psychostimulants (Sajja et al., 2016), which most likely favours the entry of cytokines from the periphery into the brain parenchyma (Croese et al., 2021; Salvador et al., 2021).

In addition to psychostimulants, such as amphetamine, cocaine, methamphetamine, and other amphetamine-like agents, we also should notice the alcohol use disorder, which represents a major global public health and social challenge that is associated with substantial morbidity and mortality. Chronic and excessive alcohol consumption can result in both reversible and irreversible cognitive deficits and is linked to structural alterations in the brain (Clergue-Duval et al., 2022; Karoly et al., 2024). Moreover, alcohol abuse may also affect immune function (Fox et al., 2017; Rutt et al., 2024) and increase susceptibility to several malignancies.

With a primary focus on the function of anti-inflammatory drugs, we present the anti-addiction effects produced by blocking the effects of neuroimmune signalling. The development of innovative, all-encompassing therapeutic approaches to address this global epidemic will be facilitated by an understanding of the critical role that neuroimmune signalling plays in drug addiction.

## 3 Neuroimmune signalling

Immunocompetent cells that interact with the peripheral immune system also cause neuroinflammation in the CNS in response to damage. For instance, peripheral immune cells may enter the CNS in response to damage (Kipnis, 2016) or stress. One preclinical study revealed that after prolonged foot shock stress, murine hippocampal bone marrow-derived monocytes infiltrate the brain (Brevet et al., 2010). Remarkably, the monocytes in this study were Iba-1-positive but glial fibrillary acidic protein (GFAP)-negative, and they acquired traits similar to those of microglia, such as a ramiform shape. Another study investigating a stress paradigm in mice revealed that bone marrow-derived monocytes infiltrate the paraventricular nucleus of the hypothalamus (Ataka et al., 2013), indicating that the activation of peripheral cells is a crucial mechanism that responds to stress (Ménard et al., 2017).

Because the functions of the central and peripheral immune systems overlap significantly, neuroimmune signals such as cytokines and neurotrophic factors are not unique to either system (Hohlfeld et al., 2007). The neurotrophic factor protein family is essential for the healthy development of the CNS in vertebrates. These variables control plasticity, dendritic arborization, and neuronal survival (Batchelor et al., 1999; Lu, 2004). Furthermore, synaptic plasticity and immune cell functions such as migration, activation, and differentiation are significantly influenced by neurotrophic factors, such as brain-derived neurotrophic factor (BDNF) and glial cell line-derived neurotrophic factor (GDNF) (Hohlfeld et al., 2007; Thoenen, 1995).

## 4 Cytokines and chemokines

In addition to contributing to neuroinflammation, neuronal activity, neuron–glia communication, neuroendocrine interactions, neurogenesis, and CNS development, cytokines and chemokines are crucial elements of the CNS neuroimmune system (Bachtell et al., 2017; Boulanger, 2009). Both the peripheral nervous system and CNS use chemokines, which are chemoattractant cytokines belonging to a family of low-molecular-weight proteins (Mélik et al., 2015; Rostène et al., 2007). These chemokines mediate their effects through seven-transmembrane G protein-coupled receptors (GPCRs) called CR1, CCR1–11, CXCR1–5, or CX3CR1 and are divided into four subfamilies according to the location of cysteine residues within the amino-terminal region: C, CC, CXC, and CX3C chemokines (Murphy, 2002; Murphy et al., 2000). The brain secretes numerous chemokines, such as CCL2, CCL3, CCL5, CXCL1, CXCL8, CXCL12, and CX3CL1 (the letter “L” represents ligand) (Bajetto et al., 2001; Tashiro et al., 1993). The hippocampus, cerebral cortex, amygdala, thalamus, and basal ganglia are among the brain areas that express the chemokine receptors CCR1, CCR4, CCR5, CCR9, CXCR2, CXCR4, and CX3CR1 (Bajetto et al., 1999; Gabuzda et al., 1998). These GPCRs can communicate via Gαi/o proteins, which inhibit adenylate cyclase activity and reduce protein kinase activity (Zheng et al., 1999). Moreover, GPCRs can increase intracellular Ca^2+^ levels and protein kinase C levels via the phospholipase C pathway through the Gq protein (Calì and Bezzi, 2010; Meucci et al., 1998).

During neuroinflammation, cytokines can exert beneficial or detrimental effects. For example, IL-37 has been reported to inhibit inflammation both *in vitro* and *in vivo*. It is a potent endogenous anti-inflammatory member of the IL-1 family. IL-37 suppresses the expression of other inflammatory cytokines, thus inhibiting disease progression (Boraschi et al., 2011). In an *in vitro* experiment, an siRNA targeting IL-37 (siIL-37) was transfected into human PBMCs stimulated with LPS. After IL-37 expression was specifically silenced, the production of IL-6 and other cytokines increased in a dose-dependent manner (Cavalli and Dinarello, 2018), indicating that IL-37 can inhibit the inflammatory effect of IL-6 and play an anti-inflammatory role. Numerous inflammatory conditions, including asthma, fibromyalgia, and chronic inflammatory illness, have been treated using pharmacotherapeutic agents targeting IL-37 (Bai et al., 2020; Meng et al., 2019), but this approach has not yet been investigated in relation to drug addiction.

Although the exact mechanism through which IL-37 reduces inflammation is unknown, it is thought to occur by blocking mammalian target of rapamycin (mTOR (Li et al., 2017)) or by inhibiting inflammasome activation (Moretti et al., 2014). Addiction processes have been linked to the mTOR pathway, particularly in relation to the effects of psychostimulants (Bailey et al., 2012; Ucha et al., 2020). On the other hand, proinflammatory cytokines are pleiotropic, meaning that they can cause 1 cell type to proliferate while simultaneously inhibiting the growth of another cell type. Tissue-invading leukocytes in the CNS release proinflammatory cytokines in response to tissue damage or neurodegeneration. Additionally, glial cells contain cytosolic multiprotein complexes called inflammasomes that activate proinflammatory caspases (mainly caspase 1 (Rathinam et al., 2012)). Methamphetamine exposure has been shown to activate inflammasomes in the context of addiction (Xu et al., 2018; Zhao et al., 2019). An inflammatory reaction and the release of proinflammatory cytokines result from the activation of these caspases. Notably, the timing of the administration of certain mediators during the disease course can affect their contribution to neuroinflammation. Fractalkine (also known as CX3CL1) has been shown to mediate nicotine withdrawal-induced hyperalgesia in rats, providing evidence for this phenomenon in the context of addiction. Finally, fractalkine can function as a neuronal off-signal to preserve the anti-inflammatory state of microglia (Ding et al., 2015).

Interestingly, the effects of amphetamine and cocaine on the anti-inflammatory cytokine IL-10 are more variable in drug addiction such that amphetamine and cocaine robustly decrease IL-10, whereas methamphetamine does not have this effect (Ligeiro de Oliveira et al., 2012; Moreira et al., 2016). Heroin and morphine can also increase the serum levels of the anti-inflammatory cytokines IL-10 in mice (Pacifici et al., 2000). The mounting evidence linking inflammatory processes to addiction is leading to the emergence of anti-inflammatory agents (IL-10) as potential new treatments for drug addiction.

In one study, the plasma CCL11 concentration was much lower in women with alcoholism than in men (García-Marchena et al., 2016). Experiments were conducted in adolescents (humans and mice) by Pascual et al. (2017), who also reported that women are more vulnerable to the inflammatory effects of ethanol overconsumption than men are: at equivalent blood alcohol levels, the plasma levels of cytokines and chemokines (IFN-γ, IL-10, IL-17A, IL-1β, IL-2, IL-4, IL-6, IL-8, CX3CL1, CCL2, and CCL3) were higher in adolescent women than in adolescent men after severe alcohol intoxication. Thus, in addition to the structural and functional sex differences in the effects of ethanol on the brains of adolescents (Mineur et al., 2022), new evidence suggests the existence of sex differences in the immune and neuroimmune responses induced by ethanol (Cruz et al., 2023). Taken together, the evidence above implicated that targeting neuroimmune signaling may be a viable stragy for treating alcohol addiction, and more research is needed to understand sex-specific differences in alcohol drinking and neuroimmune mechanisms.

## 5 Neuroimmune signalling and its role in addiction

**FIGURE 2:**
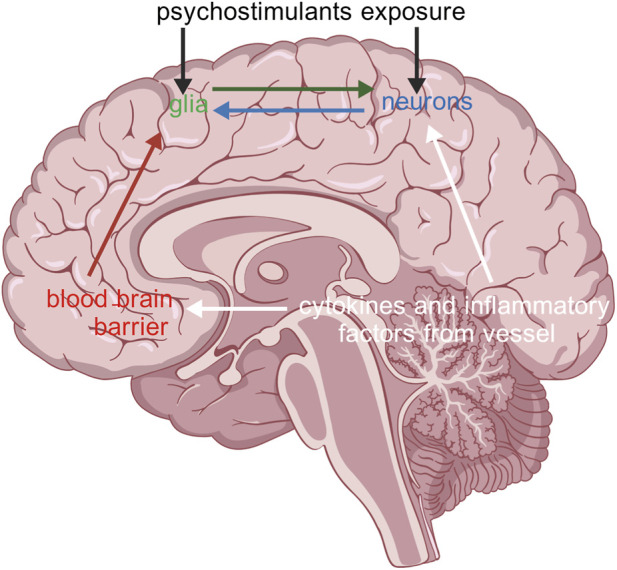
Schematic of major postulated immune interactions within the brain of individuals with drug addiction. The four main interactive compartments affected by drug addiction can be summarized as follows: 1) neuronal populations in different parts of the brain, 2) central glia that can functionally interact with neurons, 3) changes in the blood–brain barrier structure and function, resulting in pathological permeability to peripheral inflammatory cytokines, and 4) changes in peripheral immune cells and their release of soluble mediators, including cytokines.

An increasing number of studies have indicated that chemokine systems affect physiological circuits, including dopaminergic and possibly glutamatergic circuits, which underpin drug addiction, although the primary roles of chemokines were initially believed to be restricted to chemotaxis and neuroinflammation (Cui et al., 2014) (Figure 2). One chemokine system of particular relevance is the chemokine ligand/receptor pair CXCL12/CXCR4. One of the rare chemokines in the brain, CXCL12, is released by both neuronal and nonneuronal populations (Guyon, 2014). CXCL12 binds to and activates at least two receptors, CXCR4 and CXCR7; CXCR4 is the primary CXCL12 receptor in the brain and is expressed by neurons, astroglia, and microglia (Bleul et al., 1996). Dopaminergic neurons in the substantia nigra (Banisadr et al., 2002) and GABAergic MSNs in the lateral shell of the NAc (Trecki et al., 2010) express CXCR4 receptors. The FDA has approved AMD3100 (plerixafor), a CXCR4 antagonist that exhibits selectivity for CXCR4 over other chemokine receptors and can be used to study receptor function (Wilson et al., 2011).

Research has indicated that cocaine use disorder is associated with the expression of the CX3CR1 receptor. The interaction between CX3CR1 and its ligand, fractalkine (CX3CL1), facilitates microglial activation and provides neuroprotection (Ransohoff et al., 2007). Furthermore, cocaine dependence in the rat hippocampal region after social defeat stress has been linked to the interaction between fractalkine and CX3CR1 (Montagud-Romero et al., 2020). Following cocaine-induced CPP exposure, the p-p65/p65 NF-κB ratio and pCREB/CREB ratio increase in mice, but these changes are reversed in CX3CR1-KO mice (Montagud-Romero et al., 2020). Although more investigations are needed to determine the role of CX3CR1 in other drug-seeking behaviours, our results suggest that it plays a role in the activation of transcription factors that influence CPP development. A low AMPA/NMDA ratio was detected in the CX3CR1-KO mice during development; however, no change in the protein expression of glutamatergic receptor subunit was observed in these mice. These findings raise the question of whether CX3CR1 is involved in glutamate signalling. Moreover, sex-linked variations in the way in which CX3CR1 modulates the inflammatory response have been reported; female CX3CR1-KO mice responded to diet-induced inflammation through WT “male-like” microglial activation (Dorfman et al., 2017).

Substantial evidence indicates that the prevalence and course of cocaine use disorder differ by sex (Quigley et al., 2021). Men are more likely to start using cocaine, whereas women are more likely to become addicted, have stronger cravings, and relapse because of stress (Quinones-Jenab and Jenab, 2012; Winhusen and Lewis, 2013). These variations may be explained by sex-specific patterns in the CX3CL1/CX3CR1 signalling pathway, which are fuelled by neuroinflammatory mechanisms. Notably, a recent study showed that rats exposed to allopregnanolone, a neurosteroid with anti-inflammatory qualities, exhibited sex-based differences in CX3CL1 regulation (Balan et al., 2024). Additionally, after adolescent stress and alcohol exposure, CX3CR1-deficient mice displayed maladaptive coping behaviours and increased neuroinflammatory responses, underscoring the crucial role of this receptor in regulating stress-related neuroimmune pathways and susceptibility to substance use disorders (Medina-Vera et al., 2025). These results suggest that compared with males, females may have different neuroinflammatory responses, as cocaine affects neuroinflammation and CX3CL1 levels. Taken together, these studies provide a basis for additional research on the differences in drug addiction-induced neuroinflammation between the sexes and may suggest sex-specific research avenues for the development of pharmacotherapeutics to treat drug addiction.

Because the BBB controls the flow of macromolecules, ions, and neurotransmitters from the blood into the brain, it is essential for maintaining homeostasis (Erickson and Banks, 2018). Notably, the BBB helps maintain a stable milieu for optimal neuronal activity and restricts the entry of neurotoxic chemicals from the periphery to prevent serious CNS injury (Abbott et al., 2010). According to recent studies, drugs of abuse change tight junction (TJ) development and protein expression, which results in BBB malfunction (Abbott et al., 2006).

Continuous cocaine administration has been reported to increase BBB permeability by 50%. This increased permeability is accompanied by a decrease in transendothelial electrical resistance (TEER) due to a disruption of the basement membrane and neurovascular capillaries caused by the upregulated expression of TNF-α and matrix metalloproteinases (MMPs) (Sharma et al., 2009). Furthermore, cocaine transit across the BBB is characterized by TJ protein loss and modification, particularly decreased JAM-2 and zonula occludens-1 (ZO-1) levels (Dietrich, 2009). Chronic cocaine administration increases BBB permeability in the striatum and hippocampus of rats, indicating that glial and cytokine migration may affect the hippocampus without significantly altering cortical or cerebellar permeability (Davidson et al., 2018). Additionally, in free-moving rats, acute cocaine administration has been shown to modify BBB permeability and perhaps exacerbate neurotoxicity (Barr et al., 2020).

According to other studies, cocaine causes neuroinflammation and disrupts the BBB by activating microglia in the brain to release various cytokines, chemokines, and other neurotoxic substances (Buch et al., 2012). Proinflammatory cytokines are upregulated in both intracellular and extracellular compartments when pericytes are exposed to cocaine, according to earlier *in vitro* research. Furthermore, cocaine increases CXCL10 secretion by activating the Src–PDGFR-b–NF-kB pathway. Increased neuroinflammation in human brain vascular pericytes results from the activation of this pathway, which in turn promotes the disruption of neurovascular units and immune cell transmigration across the BBB (Niu et al., 2019; Sil et al., 2019).

## 6 Emerging therapies that inhibit neuroimmune signalling to treat drug addiction

### 6.1 IL-10

Anti-inflammatory drugs are emerging as possible novel treatments for drug addiction because of the accumulating evidence that inflammatory processes are linked to addiction. Research has shown that by preventing morphine-induced glial activation inside the NAc, the anti-inflammatory factor IL-10 can reduce morphine-seeking behaviour (Schwarz et al., 2011). Plasmid DNA expressing IL-10 (pDNA-IL-10) has been shown to significantly increase the expression of IL-10 mRNA *in vitro*, especially in the CD11b^+^ microglial population (Lacagnina et al., 2017). Interestingly, pDNA-IL-10 has been shown to decrease opioid self-administration when it is administered directly into the NAc of rats (Lacagnina et al., 2017), suggesting the capacity of NAc to produce IL-10 as a drug addiction cure. Xalud Therapeutics is a viral gene therapy firm that resolves the proinflammatory microglial state by driving IL-10 synthesis *in vivo* via a nonviral plasmid delivery strategy (Bachtell et al., 2015). Currently, this approach is used to treat animal models of neuropathic pain (Dengler et al., 2014), multiple sclerosis (MS) (Kwilasz et al., 2021), amyotrophic lateral sclerosis (ALS) (Gravel et al., 2016), and osteoarthritic joints to reduce discomfort from inflamed peripheral locations (Watkins et al., 2020). With respect to alcohol addiction, some studies have shown decreased IL-10 levels in the central amygdala (CeA) following chronic alcohol exposure and revealed that IL-10 exposure attenuated the effect of alcohol on inhibitory neurotransmission (Patel et al., 2021). Other studies have shown that one or three treatment cycles do not affect IL-10 levels in the CeA; however, these levels decrease in the basolateral amygdala (Marshall et al., 2017). Therefore, the role of IL-10 may depend on the type of alcohol exposure and may be more evident in the basolateral amygdala than in the CeA. This strategy has not yet been used to treat addiction, but preventing the neuroinflammatory reactions induced by activated microglia may open the door to treating neurological disorders, such as opioid and psychostimulant addiction.

### 6.2 Ibudilast

Ibudilast is a small chemical with immunomodulatory and neuroprotective properties that is frequently used in Asia to treat poststroke vertigo and asthma. Because of its intricate mode of action, it inhibits innate immune signals and penetrates the BBB (Yamazaki et al., 2011). By blocking important molecules that regulate inflammation, such as PDE, TLR4, and macrophage migration inhibitor (MIF), ibudilast exerts neuroprotective and immunomodulatory effects. Ibudilast has been shown to decrease the overproduction of proinflammatory factors by specifically targeting the TLR4 signalling pathway (Li et al., 2021). By decreasing NO, ROS, IL-1β, IL-6, and TNF-α levels and increasing the synthesis of the anti-inflammatory cytokine IL-10, ibudilast dose-dependently prevents microglial activation (Babu et al., 2021). Additionally, *in vitro* research has shown that ibudilast reduces the expression of the astrocyte and microglial activation markers GFAP and CD11b, thereby attenuating morphine and methamphetamine addiction (Beardsley et al., 2010; Schwarz et al., 2011). Furthermore, ibudilast decreases both male and female rats’ behavioural sensitivity to cocaine (Poland et al., 2016). Notably, ibudilast has shown encouraging efficacy in human clinical trials. In a recent human trial, ibudilast decreased methamphetamine use and cravings (Worley et al., 2016). By modifying microglial activity, ibudilast reduces some subjective assessments of opioid withdrawal symptoms in volunteers who are opioid dependent but is well tolerated, without significant side effects (Cooper et al., 2016). Furthermore, a randomized clinical investigation revealed that ibudilast reduces the acute proinflammatory effects of methamphetamine on people with methamphetamine use disorder (Li et al., 2020). The CNS-directed anti-inflammatory properties of ibudilast suggest that it may be used to treat opioid use disorder and addiction to other substances, such as methamphetamine.

With respect to alcohol use disorder treatment, after an infusion of alcohol, ibudilast did not affect the subjective response to alcohol craving, stimulation, sedation, or mood (Ray et al., 2017). In stress-induced alcohol craving, ibudilast promoted faster recovery to a positive mood compared with the placebo, although the difference was not significant (Ray et al., 2017). Studies have shown that participants treated with ibudilast have significantly lower levels of both peripheral and central neuroimmune markers than those treated with a placebo (Grodin et al., 2022). Overall, ibudilast modulates the relationship between mood and alcohol craving and consumption, suggesting that neuroimmune signalling may be an important target for treatment development, but increased selectivity is needed.

### 6.3 Minocycline

The most lipid-soluble tetracycline derivative that can pass through the BBB is minocycline, a semisynthetic tetracycline analogue that is also referred to as 7-dimethylamino-6-dimethyl-6-deoxytetracycline. Minocycline, like ibudilast, has immunomodulatory and neuroprotective properties in addition to its antibacterial effects. Minocycline works at the molecular level by blocking nuclear NF-κB translocation, which prevents microglial activation (Kobayashi et al., 2013). Minocycline also inhibits the activities of PKC (Nikodemova et al., 2007), P38 (Yoshida et al., 2020) and c-Jun N-terminal kinase (JNK) (Nikodemova et al., 2006). Consequently, the overproduction of inflammatory cytokines is suppressed. According to the results of animal studies, minocycline can reduce the development of tolerance and CPP induced by morphine and methamphetamine. This effect is mediated by the suppression of microglial activation (Arezoomandan and Haghparast, 2016). Additionally, minocycline inhibits methamphetamine-induced microglial activation and reduces methamphetamine self-administration and methamphetamine-induced dopamine release (Hashimoto et al., 2013; Snider et al., 2013). In rats, morphine-dependent effects have been shown to increase the expression of glutamate transporter-1 (GLT-1) (Arezoomandan et al., 2019) and reduce the likelihood of cocaine self-administration caused by long-term restraint stress through the restoration of GLT-1 expression in the NAc core (Avalos et al., 2022). However, outcomes from research on humans have been mixed. Minocycline did not significantly affect experimental pain or addiction-related outcomes in a double-blind, randomized human laboratory investigation (Arout et al., 2019). Overall, minocycline shows promise for therapeutic use in attenuating addictive behavioural reactions linked to drug consumption; nevertheless, more research in human subjects is necessary to confirm its potential.

In a double-blind, placebo-controlled trial, even though minocycline administration did not result in many side effects, the participants did not feel any change in the subjective response to alcohol or alcohol-induced craving after treatment with two different doses of minocycline for 10 days (Petrakis et al., 2019).

### 6.4 Dizocilpine

As a noncompetitive antagonist of NMDARs, dizocilpine (MK-801) efficiently suppresses the production of proinflammatory cytokines. According to *in vitro* research, dizocilpine can reduce the levels of proinflammatory factors, including COX-2 and TNF-α, which in turn can decrease microglial activation and neurotoxicity (Li et al., 2009). Additionally, in cathepsin C-treated microglia, dizocilpine reverses PKC, p38, IκBα, and p65 phosphorylation, thereby reducing neuroinflammation (Liu Q. et al., 2019). Furthermore, by blocking pERK, pSTAT3, and pAKT signalling pathways, dizocilpine prevents TNF-α and IL-6 production in mice (Liu S. et al., 2019). Dizocilpine has shown impressive efficacy in controlling drug addiction behaviour. Dizocilpine reduces methamphetamine-induced microglial activation in the striatum *in vivo* (Thomas and Kuhn, 2005). Moreover, dizocilpine decreases opioid tolerance, in part by blocking the activation of NMDARs mediated by the JNK pathway, which has been linked to the development of opioid tolerance (Guo et al., 2009). Subsequent research has shown that the antibiotic ceftriaxone (25 mg/kg, intraperitoneal injection) combined with low-dose dizocilpine (0.05 mg/kg, intraperitoneal injection) efficiently reduces morphine-induced CPP behaviour and completely prevents morphine reinstatement (Fan et al., 2012). Furthermore, dizocilpine prevents mice from acquiring cocaine-associated memory. In conclusion, dizocilpine, a very small anti-inflammatory chemical, may be used to treat drug addiction.

A number of anti-inflammatory substances, such as minocycline, amlexanox, ibudilast, and ampremilast, exhibit encouraging outcomes in rodent models, and human testing is currently in progress. Dosage is one of the many details that must be carefully considered when translating possible pharmacotherapies from animal models to humans. Its success may be impacted by the fact that the highest authorized fenofibrate dose utilized in the human clinical study was less than the level identified to decrease drinking in mice. Fenofibrate is peroxisome proliferator-activated receptor alpha (PPAR-α) agonists (Schoonjans et al., 1996), actively elevating the oxidation rate of fatty acids, and are commonly employed to treat hypertriglyceridemia (Cignarella et al., 2006). Several studies have shown that fenofibrate administration to rodents that consume alcohol voluntarily leads to a reduction in alcohol intake (Blednov et al., 2016; Haile and Kosten, 2017). However, the effective dose of apremilast that reduced ethanol preference and consumption in mice is about the same as the amount being evaluated in people (Blednov et al., 2018), indicating its translational potential for alcohol use disorder could be higher. Additionally, given the immunological dysregulation already present in patients with alcohol use disorder, possible pharmacological side effects that could trigger immune signaling should be taken into account.

Furthermore, given the polymorphisms in P2X7Rs in treatment outcomes for mood disorders in patients with comorbid alcohol use disorder, further investigation is required to determine the genetic factors underlying drug-induced immune activation in comorbid patients (Soronen et al., 2011). Additionally, the etiology of alcohol consumption disorder is similar to that of other alcohol use disorders, frequently involving disruption of the same neurotransmitter systems and brain circuits (Koob and Volkow, 2016). Similar targets for various substances of abuse may be found through overlapping mechanisms of glial activation and neuroimmune regulation of behavior. Additive effects on neuroimmune activation can also be caused by drugs of abuse (Alshehri et al., 2017; Althobaiti et al., 2016), and treatment efficacy may depend on the particular substances abused (Sari et al., 2016). Given that polydrug abusers account for the majority of drug-related deaths globally, this raises significant issues. To clarify appropriate treatment approaches, improved preclinical models and additional clinical research on co-occurring mental and drug use problems will be crucial.

Finally, therapeutic approaches based on cytokines and chemokines differ greatly from more traditional approaches that target receptors, transporters, and enzymes that support the biological actions of well-known neurotransmitters such as dopamine, glutamate, acetylcholine, and serotonin. Targeting cytokine and chemokine systems may have the advantage of preventing the rewarding and reinforcing effects of several classes of drugs of abuse, although this effect is merely hypothetical. Determining how particular cytokine and chemokine receptor antagonists impact opioid side effects such as dependency, relapse, constipation, and respiratory depression will be crucial in future research. In conclusion, these recent developments are encouraging for the development of pharmacotherapies based on cytokines and chemokines for the treatment and prevention of drug use disorders (Ahearn et al., 2021).

## 7 Conclusion

In addition to neuroinflammation and chemotaxis, cytokines and chemokines play key roles in the control of the physiological systems that underpin addiction. In the current review, we discuss how these incredibly adaptable inflammatory proteins help the immune system and interact when drug addiction is present. We also reviewed cytokine- and chemokine-based anti-inflammatory treatment approaches and critically examined the cytokine and chemokine processes underlying substance use disorders.

Accumulating evidence has shown that neuroimmune signalling pathways can respond to psychoactive drugs and cause neuroinflammation and long-lasting immune responses, which can lead to addiction. Additionally, the reactions of the neuroimmune system, including dynamic and well-coordinated signal transduction at several levels, are intricate. Therefore, a better understanding of the underlying mechanisms is needed to elucidate how drug-induced changes in neuroimmune signalling pathways contribute to the development of addiction. Crucially, the development of successful treatments depends on an understanding of the complex signalling characteristics of neuroimmune cells within the larger framework of the neuroimmune system.

The described biomarker profile has also been consistently linked to anxiety and depression symptoms in people with drug addiction, indicating that an increased affective symptom burden is associated with increased inflammatory signalling and decreased neurotrophic support. In particular, anxiety/depression scores were negatively correlated with BDNF levels and positively correlated with TNF-α and IL-18 levels (Decker Ramirez and Schank, 2026; Kazmi et al., 2022). These results imply that lower levels of neuroplasticity-related biomarkers and a more prominent proinflammatory environment may be linked to increased emotional symptomatology. These associations suggest the plausibility of an inflammation–affect relationship as part of the clinical profile of drug users, although causation cannot be established (Decker Ramirez and Schank, 2026; Moura et al., 2022). Reduced neurotrophic signalling may co-occur with affective symptoms, according to its inverse relationship with BDNF. This pattern aligns with the correlational structure reported by Tyler et al. (Tyler et al., 2025), in which greater clinical severity was associated with higher IL-18 and TNF-α levels and lower BDNF levels (Decker Ramirez and Schank, 2026; Kazmi et al., 2022). Overall, our findings highlight the clinical significance of anxiety and depression as components of the larger biological profile of individuals with drug addiction (Gellé et al., 2024; Shafiee et al., 2023).

Finally, the differences in the functions of neuroimmune signalling pathways that target certain brain regions or neural circuits in individuals with addiction are another crucial factor to consider. Region-specific cytokine and chemokine modifications are needed to address this issue and identify their unique roles in addiction. Furthermore, pharmacological and genetic strategies targeting neuroimmune signalling may be promising for future drug addiction treatment. In conclusion, the development of addiction is strongly influenced by neuroimmunopharmacology mediated by cytokines and chemokines. The development of innovative treatments to overcome drug addiction requires a better understanding of the interactions between neuroimmune signalling, neuroinflammation, and drug addiction.
